# Automated Neuromuscular Assessment: Machine-Learning-Based Facial Palsy Classification Using Surface Electromyography

**DOI:** 10.3390/s26010173

**Published:** 2025-12-26

**Authors:** Ibrahim Manzoor, Aryana Popescu, Sarah Ricchizzi, Aldo Spolaore, Mykola Gorbachuk, Marcos Tatagiba, Georgios Naros, Kathrin Machetanz

**Affiliations:** Department of Neurosurgery and Neurotechnology, Eberhard Karls University, Hoppe-Seyler-Straße 3, 72076 Tübingen, Germanygeorgios.naros@med.uni-tuebingen.de (G.N.)

**Keywords:** facial palsy, electromyography, machine learning, random forests, decision trees

## Abstract

**Highlights:**

**What are the main findings?**

- Time-domain EMG features during facial movements effectively reflect facial nerve dysfunction.
- Ensemble ML models achieved up to ~84.8% accuracy in automated HB classification from EMG data in facial palsy.

**What are the implications of the main findings?**

- EMG-based ML enables objective, non-invasive assessment of facial palsy severity.
- This method can enhance diagnostic consistency and enable longitudinal monitoring in both clinical practice and research settings.

**Abstract:**

Facial palsy (FP) impairs voluntary control of facial muscles, resulting in facial asymmetry and difficulties in emotional expression. Traditional assessment methods to define the severity of FP (e.g., House–Brackmann score, HB) rely on visual examinations and, therefore, are highly examiner-dependent. This study proposes an alternative approach using facial surface electromyography (EMG) for automated HB prediction. Time-domain EMG features were extracted during different facial movements (i.e., smile, close eyes, and raise forehead) and analyzed through nine different machine learning (ML) models in 58 subjects (51.98 ± 1.67 years, 20 male) with variable facial nerve function (HB 1: n = 16, HB 2–3: n = 32; HB 4–6: n = 10). Model performances were evaluated based on accuracy, precision, recall, and F1-score. Among the evaluated models, ensemble-based approaches—particularly a random forest model with 100 trees and a decision tree ensemble—proved to be the most effective with classification accuracies ranging from 81.7 to 84.8% and from 81.7 to 84.7%, depending on the evaluated facial movement. The results indicate that ensemble-based ML models can reliably distinguish between different FP grades using non-invasive EMG data. The approach offers a robust alternative to subjective clinical scoring, potentially improving diagnostic consistency and supporting longitudinal monitoring in clinical and research applications.

## 1. Introduction

Facial palsy (FP) is the most common cranial nerve dysfunction that leads to partial or complete loss of voluntary muscle movement, causing functional and esthetic impairments that severely impact quality of life (QoL) [1]. The House–Brackmann (HB) grading system is a widely used clinical tool for assessing the severity of FP [2,3]. Despite its clinical utility, the HB score has notable limitations: it relies heavily on visual inspection and clinician judgment, making it prone to subjectivity and inter-rater variability [4].

Recent years have witnessed a surge in image- and video-based facial analysis, which enables objective quantification of visible facial motion. These approaches have been applied to measure facial symmetry and expression dynamics, showing strong potential for standardized, reproducible grading of FP [5,6]. However, these methods typically assess visible outcomes of muscle activity rather than the underlying neuromuscular function. Electromyography (EMG), by contrast, directly measures electrical signals produced during muscle activation and provides complementary functional information about neuromuscular status, including subtle asymmetries that may not be apparent on visual inspection [7]. Therefore, it plays a pivotal role in diagnosing neuromuscular disorders, evaluating muscle fatigue, monitoring rehabilitation, and other applications [8,9,10,11,12,13]. Surface EMG (sEMG) provides a non-invasive means of acquiring these signals, making it suitable for routine clinical use and longitudinal assessments [14].

Advances in machine learning (ML) and biomedical signal processing have enabled EMG-based automated classification in various neuromuscular applications, including hand-movement recognition and detection of diseases such as amyotrophic lateral sclerosis [15,16,17]. Building on these advances, the integration of EMG signal analysis with ML-based classification approaches offers considerable potential for objective assessment of FP. While initial studies primarily focused on correlating EMG data with established grading scales [18,19,20], Holze et al. [21] proposed a semi-automated sEMG-ML approach as an objective alternative to HB grading and demonstrated encouraging classification accuracies. However, as only three machine-learning approaches were evaluated in a cohort of 28 patients, the generalizability of the findings remains limited.

Against this background, our proof-of-concept study investigates the effectiveness of nine machine learning models applied to surface EMG data for the classification of FP. This comprehensive evaluation aims to advance the development of objective, data-driven methods for FP grading, which may reduce subjectivity and enhance clinical decision-making.

## 2. Materials and Methods

### 2.1. Study Cohort

This prospective pilot study enrolled 51 patients with varying grades of facial function, as well as a control group of 7 healthy individuals, to investigate various ML techniques in assessing the FP severity on sEMG. The patients were recruited during outpatient (i.e., skull base and facial nerve consultation) and inpatient stays in our department. Patients with bilateral FP or incomplete sEMG data were excluded. Detailed cohort characteristics are presented in Table 1. The study was approved by the local ethics committee of Eberhard Karls University of Tübingen and conducted in accordance with the Declaration of Helsinki. Ethical considerations included obtaining informed consent from all participants.

### 2.2. Data Collection and Study Design

**Figure 1:**
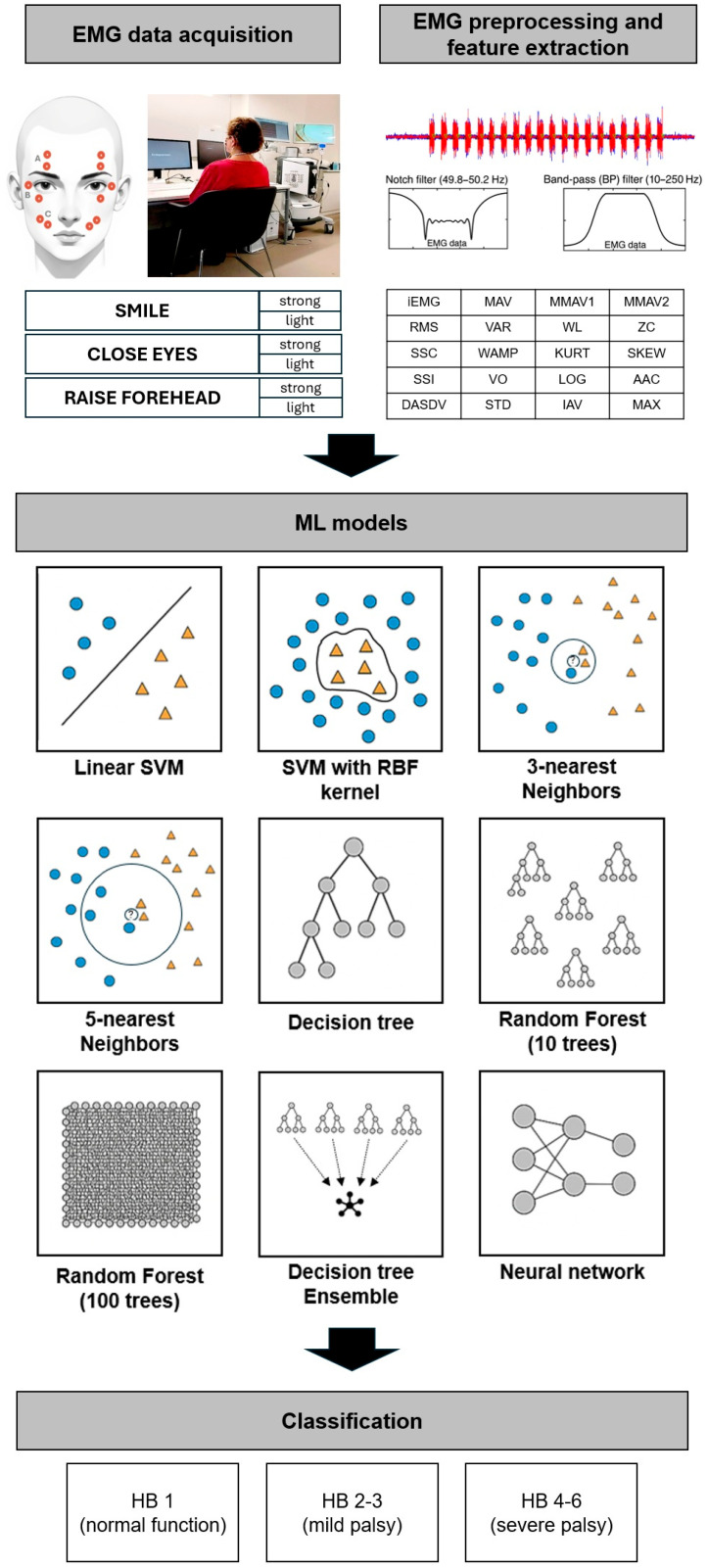
**Study design and data analysis pipeline** demonstrating the electrode mounting (top left), time series features of surface electromyography (top right), machine learning models (middle), and classification of facial palsy severity (bottom). AAC: Average amplitude change, DASDV: Difference absolute standard value, HB: House–Brackmann score, IAV: Integral of absolute value, iEMG: Integrated EMG, KURT: Kurtosis, LOG: Log detector, MAV: Mean absolute value, MAX: Maximal amplitude, MMAV1: Modified mean absolute value 1, MMAV2: Modified mean absolute value 2, RMS: Root mean square, SKEW: Skewness, SSC: Slope sign change, SSI: Simple square integral, STD: Standard deviation, SVM: Support vector machine, VAR: Variance, VO: V-order, WAMP: Willison amplitude, WL: Waveform length, ZC: Zero crossing.

The FP severity was classified by the HB score, which categorizes facial function into ranges from 1 (normal) to 6 (total paralysis) based on different facial movements (e.g., eye closure) [3]. The grades of palsies were assigned by two experienced examiners, and differences were resolved in consensus discussion to prevent variability across different examiners, which is a problem of the HB score [4]. Due to an imbalanced distribution of FP grades, we required stratified sampling for model training and, therefore, divided patients into three groups: HB 1 (no FP), HB 2–3 (mild FP), HB 4–6 (severe FP). A differentiation of individual classification of each possible HB grade (1–6) was not evaluated due to the limited sample size. After positioning the sEMG electrodes (Reusable Ag/AgCl snap electrodes with 4 mm diameter, Biopac Systems, Inc., Goleta, CA, USA) in a bipolar setting according to the scheme in Figure 1, each participant underwent a 30-min sEMG session, during which they performed six predefined facial movements targeting the zygomatic, orbicularis oculi and frontal facial muscles: smile strongly, smile lightly, close eyes strongly, close eyes lightly, raise forehead strongly, and raise forehead lightly. Each movement task consisted of 20 repetitions with the same movement sequence of a 3-s MOVE interval and a 4-s REST interval (longer relaxation duration allows muscle recovery, prevents fatigue, and ensures baseline stability). Stimuli were presented via a computer screen in front of the patients and controlled by the Psychopy^®^ software (version 2024.1.1) to ensure precise movement timing (Figure 1).

Prior to starting the experiment, subjects were instructed in detail about the meaning of the instructions. They had to perform each movement with either maximum or slight effort: strong or light smiling, full eye closure with or without squinting, and forehead raising with high or low intensity. Throughout the whole session, a trained investigator was present in the room and monitored the performance of each trial to ensure that all commands were understood and executed correctly. The EMG data were streamed with the commercial Neuro Omega V2.0 recording device (Alpha Omega Engineering, Nof HaGalil, Israel) at a sampling rate of 2000 Hz and transferred and stored on an external computer.

### 2.3. Signal Processing and Machine Learning Models

Raw EMG signals were analyzed offline using custom-written scripts in MATLAB (MathWorks Inc., Natick, MA, USA, R2022b). During preprocessing, a notch filter (49.8–50.2 Hz) was applied to eliminate powerline interference, followed by a band pass filter (10–250 Hz) to ensure a clean and physiologically relevant signal. Subsequently, 20 time series features were extracted for each individual interval and separately for each movement type. For an in-depth description of the sEMG features analyzed, please refer to the previous publication [22].

The preprocessed feature set served as input for supervised machine learning models. To obtain a broad and representative comparison of classical and modern machine learning techniques, the following nine models were examined: (1) SVM with linear and (2) radial basis function (RBF) kernels, (3 and 4) k-nearest neighbors (*k*-NN; k = 3 or 5), (5) a decision tree (DT) classifier, (6 and 7) random forest models (RF; 10 and 100 trees), (8) a DT ensemble (DTE), and (9) a neural network (NN) (Figure 1). All models were implemented and tested using MATLAB-integrated functions [23]. Model performance was primarily assessed using repeated stratified trial-level k-fold cross-validation. This approach was chosen instead of strictly subject-wise splitting because the number of subjects per HB grade was both limited and imbalanced. The dataset was split into five stratified folds (k = 5), corresponding to an 80/20 train–test ratio in each iteration. In each repetition, one fold served as the test set and the remaining four folds were used for training. This procedure was repeated 10 times, resulting in 50 independent train–test splits, implemented using MATLAB’s *cvpartition* (k-fold = 5, Stratify = true). This evaluation strategy follows established recommendations for reducing variance and bias in model selection, where k-fold cross-validation provides a good trade-off between bias and variance of the error estimate [23,24]. Hyperparameters were optimized using grid search [25].

For multiclass classification (HB 1, HB 2–3, and HB 4–6), we implemented SVMs in MATLAB using the *fitcecoc* function with *templateSVM* as the base learner. This error-correcting output codes (ECOC) framework decomposes the three-class problem into multiple binary SVMs (one-vs-one), whose outputs are combined to produce a single multiclass prediction. Within this setup, we evaluated both a linear SVM (model 1) and an SVM with an RBF kernel (model 2). These two methods differ in the way that a linear SVM uses a linear decision boundary (hyperplane) to separate the data, while an SVM with an RBF kernel uses a non-linear decision boundary by projecting the data into a higher-dimensional space. The RBF kernel is defined as follows:

$$k(x_{i},x_{j})=exp(-\frac{{\left||x_{i}-x_{j}|\right|}^{2}}{2\sigma^{2}})$$

where $x_{i}$ and $x_{j}$ are data points in the feature space, and σ is a parameter that controls the width of the Gaussian kernel. We evaluated both linear and RBF SVM classifiers and standardized all input features prior to training. A subject-wise grid search indicated that the best performance was obtained with C = 10 and automatic kernel scaling. Accordingly, the final linear and RBF SVM models both used C = 10.

The k-nearest neighbors (*k*-NN) classifier assigns each test sample to the majority class among its k closest training samples in feature space. We implemented *k*-NN using MATLAB’s *fitcknn.* A grid search guided the final setup, and we report results for k = 3 (best setting) and k = 5 using Euclidean distance.

The classification performance of a decision tree (DT) was evaluated by using *fitctree*, binary splits, and Gini impurity (model 5). This algorithm recursively divides the feature space into regions that maximize the homogeneity of the target variable within each region [26]. A grid search indicated that the best cross-validated performance was achieved with MaxNumSplits = 1, MinLeafSize = 1, and the GDI criterion, and these settings were used for all reported DT results.

Random forest classification was performed by using the *TreeBagger* function with 10 (model 6) and 100 trees (model 7). Each tree was trained on a random subset of the data using bootstrapping [27]. The final prediction was made by averaging the predictions of all individual trees for regression tasks or by majority voting for classification tasks. Bootstrap resampling and random feature subsetting at each split increased diversity between trees and therefore reduced variance of the ensemble, which was particularly beneficial given the moderate dataset size.

A DTE method with 100 trees (model 8) for FP classification was examined using *fitcensemble*, in which bagging was carried out using bootstrap aggregating. This means that each sample could contain some data points more than once and not contain others at all. A grid search showed that an ensemble of 100 pruned decision trees provided the best cross-validated performance, and this configuration was used for all reported DTE.

Finally, the MATLAB function *fitcnet* was used to implement FP classification by a feedforward, fully connected neural network (model 9). A grid search identified the best configuration as a three-layer network with 100, 25, and 10 neurons, ReLU activations, and an initial learning rate of 0.5. This architecture was used for all reported neural-network results. To reduce overfitting, we applied L2 weight regularization and allowed a high maximum number of training iterations to ensure convergence. Class labels were represented as one-hot vectors, and the network was trained using the categorical cross-entropy loss

$$L=-\sum_{i=1}^{N}\sum_{c=1}^{C}y_{i,c}\log p_{i,c}$$

where $y_{i,c}$ is the one-hot target for sample i and class c, and $p_{i,c}$ is the corresponding softmax probability.

### 2.4. Performance Ratios and Statistics

Statistical tests were performed using MATLAB (MathWorks Inc., Natick, MA, USA, R2022b). We used the following metrics to provide a comprehensive assessment of the aforementioned classification models: accuracy (ACC), precision, recall, and F1-score, which were reported averaged across all splits. Since our analysis involves a multi-class problem (HB 1 vs. HB 2–3 vs. HB 4–6), we used a one-vs-rest strategy (OvR; also referred to as one-vs-all in MATLAB documentation) to compute per-class metrics. In this formulation, each class is evaluated by treating that class as the positive category and all remaining classes as the negative category. This is the standard multiclass evaluation design used by MATLAB’s *perfcurve* and *rocmetrics* functions. To obtain overall performance, macro-averaged metrics were calculated by taking the mean of the individual OvR results across all three classes. This macro-averaging was applied to precision, recall, F1-score, and accuracy to ensure that each class contributed equally to the overall summary, regardless of class size. Furthermore, we conducted a binary classification subanalysis comparing HB 1 of healthy subjects and HB 1 of patients. The same repeated stratified 5-fold cross-validation with 10 repetitions was applied within this restricted dataset, meaning that training and test sets were re-partitioned exclusively within the HB 1 group. Stratification ensured that each fold preserved the proportion of healthy versus postoperative HB 1 cases, thereby preventing bias from potential imbalances. Finally, receiver operating characteristic (ROC) curves were generated to illustrate the model’s discriminatory ability by plotting the sensitivity (=recall) against the specificity. The area under the curve (AUC) of the ROC curve quantified the overall performance of the classifier, with a value closer to 1 indicating superior classification ability.

## 3. Results

### 3.1. Clinical Characteristics

This prospective study included 58 subjects, enrolling 42 patients with facial palsy HB grade 2–6, while 16 subjects had no FP (HB 1). The majority of FP were iatrogenic (39/42, 92.9%). The average duration of the FP was 25.24 ± 75.46 months. Surface EMG recordings were successfully conducted in all participants without any reported complications or adverse events.

### 3.2. ML-Based Facial Function Classification

**Figure 2:**
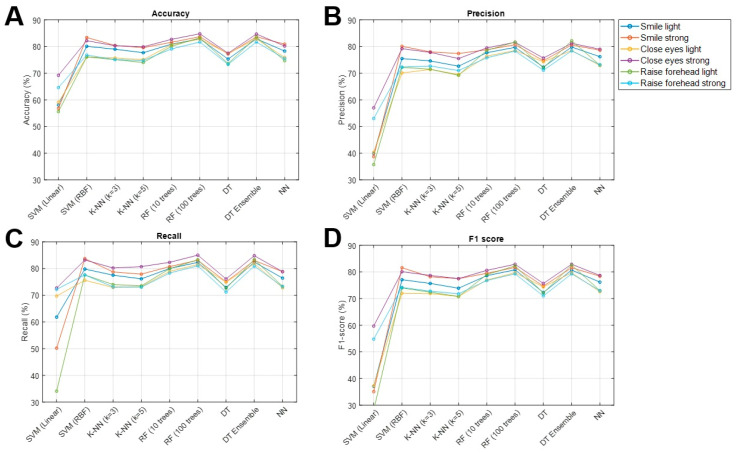
**Graphical representation of the performance metrics across all movement types (averaged across all splits).** Subplots demonstrate (**A**) the accuracy, (**B**) precision, (**C**) Recall and (**D**) the F1 score. DT: Decision tree, *k*-NN: k-nearest Neighbors, RF: Random forest, SVM: Support vector machine.

Across the six facial movements, we compared nine ML models with respect to their ability to classify the degree of facial palsy (no FP, mild FP, severe FP) from sEMG. The random forest with 100 trees (RF100, model 7) and the bagged decision-tree ensemble (DTE, model 8) achieved the strongest and most consistent performance across all six movements. For RF100, accuracy ranged from 81.7 to 84.8%, recall from 81.0 to 85.0%, precision from 78.3 to 81.7%, and F1-score from 79.3 to 82.9%. The DTE achieved very similar values, with accuracy between 81.7 and 84.7%, recall between 80.9 and 84.8%, precision between 78.4 and 82.2%, and F1-score between 79.4 and 82.9%. Performance was comparable for smiling, blinking, and forehead movements, with slightly higher accuracies for the strong movements compared to their light counterparts. Detailed results for all nine models and six movements are reported in Table 2 and Figure 2.

**Figure 3:**
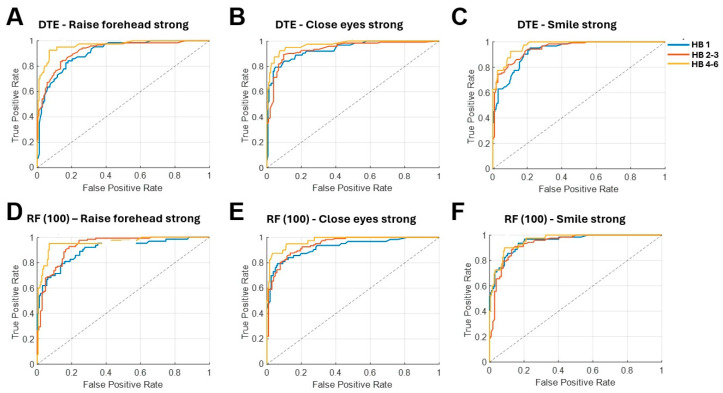
**Receiver operating characteristic (ROC) curves of the decision tree ensemble and random forest (100 trees) model** differentiating HB 1, HB 2–3, and HB 4–6. Subplots (**A**–**C**) demonstrate the ROC curves of DTE for different movements, while subplots (**D**–**F**) demonstrate the ROC curves of RF(100). DTE: Decision tree ensemble, HB: House–Brackmann score, RF: Random forest.

The ROC analysis confirmed this advantage of tree-based ensembles. For RF100, AUC values across the six movements were approximately 0.9–0.95 for HB 1, 0.92–0.94 for HB 2–3, and 0.95–0.97 for HB 4–6. The DTE showed similarly high discrimination with AUCs of about 0.91–0.95 (HB 1), 0.92–0.95 (HB 2–3), and 0.93–0.98 (HB 4–6) (Figure 3). These findings indicate that tree-based ensemble classifiers are particularly well-suited for sEMG-based FP classification.

Conversely, the linear SVM (model 1) performed the worst among all ML models, with a very poor classification performance compared to the other models. This suggests that the linear SVM struggled to effectively capture the complex patterns within the EMG data.

### 3.3. Differences in HB 1 Facial Function in Healthy Subjects and Patients

**Figure 4:**
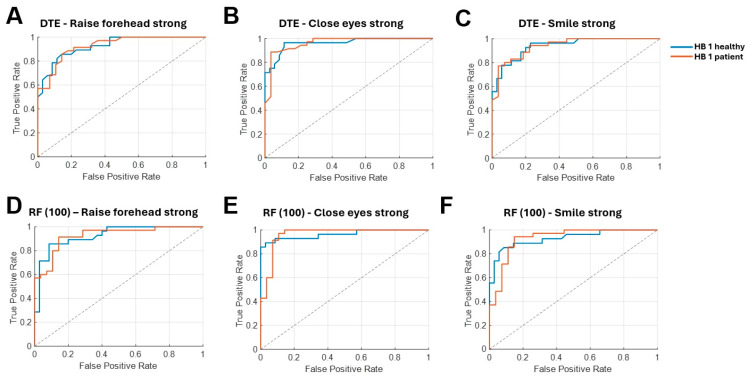
**Receiver operating characteristic (ROC) curves of the decision tree ensemble (A–C) and random forest (100 trees, D–F) model** differentiating HB 1 of healthy subjects and patients. DTE: Decision tree ensemble, HB: House–Brackmann score, RF: Random forest.

In a dedicated subcohort analysis, we examined whether patients with clinically normal facial function after surgery (HB 1 patient) could be distinguished from healthy controls (HB 1 healthy) using the RF100 and DTE models. The results demonstrated that the two groups could be reliably distinguished based on sEMG signals, with classification accuracies ranging between 85.0% and 97.2% using the RF100 model, while the DTE reached 84.0–97.2%, with the best performance consistently observed for close eyes lightly (Table 3, Figure 4). Furthermore, ROC analyses showed excellent separability for both models, with AUC values of 0.93–0.99 for HB 1 healthy subjects and 0.93–0.99 for HB 1 patients across movements. Together, these results indicate that, despite a normal HB 1 grading on clinical examination, postoperative patients exhibit systematic differences in facial EMG activity compared with non-operated individuals.

## 4. Discussion

In clinical practice, the classification of FP traditionally relies on visual inspection and manual grading systems such as the House–Brackmann (HB) scale, which are prone to significant inter-rater variability [4]. To address this limitation, the use of photographic and video-based visual assessments has been proposed [6,28,29]. However, image-based classification methods quantify only the externally visible manifestations of FP and do not provide direct information about the underlying neuromuscular activation. By contrast, sEMG captures muscle activity with high temporal sensitivity, allowing the detection of subtle differences in activation amplitude, timing, and co-activation patterns that remain below the threshold of visual inspection [18,30]. Therefore, although sEMG does not yield mechanistic insight into the pathophysiology of FP, its sensitivity to functional changes offers complementary information that may help detect subclinical dysfunction or residual activation capacity.

The potential of sEMG in FP assessment has been explored in several promising studies, supporting its physiological validity [19,20,21,31,32]. Franz et al. [31] examined patients with FP following vestibular schwannoma surgery and identified significantly greater signal variability on the affected side, with a strong correlation between sEMG measurements and the Sunnybrook grading scale. Ryu et al. [20] demonstrated that sEMG not only correlated with clinical grading systems (HB, Sunnybrook, and Yanagihara) but also showed alignment with nerve conduction studies (NCS), thus supporting its physiological validity across multiple modalities. Additionally, a semi-automated sEMG- and ML-based system developed by Holze et al. [21] demonstrated promising classification results and represented an objective alternative to the subjective HB scale. Together, these findings support the utility of sEMG as a sensitive physiological biomarker of facial nerve function.

Our findings further build on this evidence. Machine-learning models trained on sEMG data reliably distinguished among clinically relevant severity levels (HB 1, 2–3, and 4–6). Ensemble-based methods showed robust performance, which was consistent with their established strength in handling noisy biomedical signals and complex non-linear relationships. In contrast, SVMs demonstrated comparatively lower performance in our dataset. Importantly, sEMG enabled discrimination between HB 1 in healthy individuals and HB 1 in postoperative vestibular schwannoma (VS) patients, despite the absence of visible facial asymmetry. This represents a functional advantage of sEMG-based assessment: it can uncover early or subclinical physiological differences that image-based methods are generally less sensitive to. While sEMG cannot directly characterize the underlying neural pathophysiology, it provides sensitive markers of neuromuscular integrity that may indicate early dysfunction or residual activation capacity. However, the sEMG-based classifiers employed in our study should be regarded as complementary functional information to imaging modalities and clinical grading systems, rather than replacements.

The strong performance of RF models in our study aligns with a growing body of work demonstrating the suitability of tree-based algorithms for EMG-based classification tasks: Holze et al. [21] demonstrated that logistic regression and *k*-NN outperformed SVM in both the binary classification task (‘normal’ vs. ‘impaired facial function’) and the multiclass classification of HB grades 1, 2, and 3. Beyond the context of FP, numerous biomedical applications have shown that combining ML with EMG signals yields high discriminative performance. For example, Yuan et al. [33] found that RFs outperformed artificial NNs and SVMs for sEMG-based Chinese sign language classification. Gokgoz et al. [34] demonstrated that among multiple tree-based classifiers, random forests best differentiated EMG patterns in myopathy and amyotrophic lateral sclerosis (ALS). These consistent results across neuromuscular contexts underscore the resilience, noise tolerance, and generalization capability of RF models—characteristics that align with their strong performance in our FP dataset.

Beyond diagnostic classification, the physiological sensitivity of sEMG offers several potential therapeutic applications in the FP classification. The capacity to detect nuanced discrepancies—like the difference between HB 1 healthy subjects and HB 1 patients—has meaningful clinical implications. sEMG-based monitoring may help detect early neuromuscular deterioration, track recovery, or refine prognostic estimations. Biofeedback or rehabilitation systems could leverage sEMG to identify residual muscle activity—even when clinically invisible—and, therefore, enable patients to selectively activate weak muscles and optimize targeted exercises. Continuous sEMG monitoring may allow clinicians to track recovery progress objectively, adjust therapy intensity, and identify early signs of neuromuscular deterioration. In this context, previous studies have already demonstrated the utility of invasive EMG in outcome prediction [35,36]. For instance, Rampp et al. [37] developed a predictive model integrating intraoperative EMG data with preoperative HB grade and tumor size to estimate postoperative facial nerve function. Their findings highlight the multifactorial nature of FP prognosis, with tumor size being a particularly influential factor in VS cases. Building upon this, ML may offer further support in therapeutic contexts by enabling the integration of physiological data into predictive or adaptive treatment frameworks. An example of this potential is provided by Zhu et al. [38], who combined sEMG signals with electroacupuncture in a deep-learning model, illustrating how advanced signal processing can be effectively paired with innovative treatment modalities.

### Limitations

An important limitation of the study is the modest sample size, which led to class imbalance, particularly in the higher HB grades (HB 4–6). Class imbalance may bias ML models toward the majority class, leading to poor detection of infrequent but clinically relevant outcomes. Collapsing HB grades into three categories improved model stability but reduced granularity, and therefore, conclusions regarding fine-graded severity distinctions should be interpreted with caution. Furthermore, the healthy control group was younger than the patient cohort, and most FP cases were postoperative, meaning that age-related physiological differences and postoperative tissue changes could influence sEMG signals independently of nerve function. In addition, natural inter-individual asymmetry—such as variations in muscle mass, fiber orientation, or facial dominance—may affect measurements. Although recordings were standardized and supervised, sEMG remains susceptible to artifacts from movement variability, fatigue, environmental noise, or technical factors. However, the demographic and clinical characteristics of the cohort are representative of a neurosurgical FP population [39,40,41]. Furthermore, stratified sampling for model training, as well as extensive preprocessing, were performed, and all recording sessions were supervised by trained investigators. Nevertheless, normalization and preprocessing mitigate these effects but cannot eliminate them entirely. A further limitation of the present study is that the use of ML methods means that it is not possible to say exactly which of the 20 time series features were used by the models, i.e., which ones are the best. However, a previous study of ours investigated this and showed that 5/20 time series features seem to be the most appropriate [22]. Finally, long-term outcome data were not available, preventing the assessment of prognosis or recovery prediction. Future longitudinal studies with age-matched controls, non-surgical FP, and more balanced class distributions are needed to confirm generalizability and to explore the predictive potential of sEMG-based approaches.

## 5. Conclusions

This study provides an important contribution to the development of objective and automated systems for assessing facial palsy. The results highlight the potential of sEMG data, particularly in combination with ensemble-based machine learning models such as random forests, to detect subtle neuromuscular differences beyond what is visible in clinical inspection. Our findings suggest that sEMG-based approaches may support more precise grading, early detection, and individualized prognosis, offering a valuable complement to traditional image-based methods. It paves the way for real-time feedback systems that could aid healthcare professionals in monitoring patient progress, contributing to more tailored and effective treatment plans.

## Figures and Tables

**Table 1:** Cohort characteristics.

	Total N = 58	Patients N = 51	Healthy Subjects N = 7
**Age**	51.98 ± 1.67 years	54.65 ± 12.48 years	32.57 ± 15.67 years
**Gender**			
Male	20 (34.5%)	17 (33.3%)	3 (42.9%)
Female	38 (65.5%)	34 (66.7%)	4 (57.1%)
**HB Grade**			
1	16 (27.6%)	9 (17.6%)	7 (100%)
2	10 (17.2%)	10 (19.6%)	0 (0%)
3	22 (37.9%)	22 (43.1%)	0 (0%)
4	4 (6.9%)	4 (7.8%)	0 (0%)
5	4 (6.9%)	4 (7.8%)	0 (0%)
6	2 (3.4%)	2 (3.9%)	0 (0%)
**Side of FP or Surgery**			
Right	22 (37.9%)	22 (43.1%)	0 (0%)
Left	29 (50.0%)	29 (56.9%)	0 (0%)
No FP/surgery	7 (12.1%)	0 (0%)	7 (100%)
**Etiology of FP**			
Idiopathic	2 (3.4%)	2 (2.0%)	0 (0%)
Tumor	1 (1.7%)	1 (2.0%)	0 (0%)
Iatrogenic	48 (82.8%)	48 (94.1%)	0 (0%)
No FP/surgery	7 (12.1%)	0 (0%)	7 (100%)

HB: House–Brackmann; FP: Facial palsy. Surgery always means posterior skull base surgery (i.e., VS surgery or meningioma).

**Table 2:** Performance metrics across all movement types and machine learning models differentiating HB 1, HB 2–3, and HB 4–6.

Movement	Metric (%)	SVM (Lin.)	SVM (RBF)	*k*-NN (k = 3)	*k*-NN (k = 5)	RF (10)	RF (100)	DT	DT Ensemble	NN
Smile Strong	Accuracy	56.8 ± 1.7	83.4 ± 1.8	80.4 ± 2.5	79.6 ± 3.1	81.6 ± 1.9	83.7 ± 2.7	77.2 ± 3.2	83.7 ± 2.1	80.9 ± 3.1
	Precision	38.7 ± 2.4	80.1 ± 2.7	78.0 ± 3.1	77.4 ± 3.8	78.8 ± 2.6	80.7 ± 3.3	74.7 ± 3.7	80.6 ± 2.6	78.6 ± 4.5
	Recall	50.2 ± 1.4	83.7 ± 2.8	78.7 ± 3.2	77.9 ± 3.7	80.7 ± 2.2	83.1 ± 3.3	75.0 ± 3.5	83.0 ± 2.4	78.8 ± 3.5
	F1-score	35.1 ± 4.2	81.6 ± 2.4	78.2 ± 3.0	77.5 ± 3.7	79.5 ± 2.3	81.7 ± 3.2	74.7 ± 3.4	81.6 ± 2.3	78.4 ± 4.1
Smile Light	Accuracy	58.1 ± 1.5	80.1 ± 2.3	79. ± 2.7	77.7 ± 3.1	80.9 ± 2.2	82.9 ± 2.0	75.3 ± 3.1	82.9 ± 2.2	78.3 ± 2.9
	Precision	39.9 ± 2.1	75.5 ± 3.1	74.6 ± 3.0	72.63 ± 3.4	77.7 ± 2.7	79.7 ± 2.2	72.3 ± 3.75	79.8 ± 3.0	76.2 ± 3.4
	Recall	61.8 ± 8.4	79.8 ± 2.9	77.5 ± 3.2	76.12 ± 3.9	80.0 ± 2.5	82.3 ± 2.6	72.9 ± 3.7	82.4 ± 2.5	76.4 ± 3.6
	F1-score	37.1 ± 3.7	77.1 ± 2.9	75.7 ± 3.0	73.9 ± 3.6	78.6 ± 2.4	80.8 ± 2.2	72.3 ± 3.6	80.7 ± 2.6	76.2 ± 3.4
Close Eyes Strong	Accuracy	69.2 ± 2.2	82.2 ± 2.3	80.3 ± 2.4	79.9 ± 2.5	82.7 ± 2.3	84.8 ± 2.1	77.5 ± 2.8	84.7 ± 1.8	80.2 ± 2.4
	Precision	57.0 ± 3.0	79.2 ± 3.2	77.8 ± 2.9	75.5 ± 3.1	79.5 ± 3.1	81.6 ± 2.9	75.7 ± 3.2	81.2 ± 2.3	79.0 ± 2.8
	Recall	72.7 ± 4.6	83.2 ± 2.5	80.2 ± 3.0	80.7 ± 3.1	82.3 ± 2.7	85 ± 2.8	76.1 ± 3.2	84.8 ± 2.2	78.8 ± 2.7
	F1-score	59.7 ± 3.5	80.1 ± 2.7	78.7 ± 2.8	77.5 ± 3.0	80.6 ± 2.7	82.9 ± 2.6	75.7 ± 3.0	82.9 ± 2.1	78.7 ± 2.6
Close Eyes Light	Accuracy	59.1 ± 1.5	76.1 ± 2.3	75.7 ± 3.0	75.1 ± 2.8	80.7 ± 2.1	82.8 ± 2.3	77.4 ± 2.9	82.9 ± 2.2	75.9 ± 3.0
	Precision	40.3 ± 1.9	70.1 ± 3	71.4 ± 3.9	69.5 ± 3.5	76.4 ± 2.9	78.5 ± 3.0	74.3 ± 3.4	78.5 ± 2.9	72.9 ± 3.9
	Recall	69.7 ± 1.1	75.6 ± 3.4	72.9 ± 3.8	73.2 ± 3.6	78.9 ± 2.9	81.5 ± 2.9	74.9 ± 3.4	81.8 ± 2.8	72.8 ± 3.6
	F1-score	37.3 ± 3.2	72.0 ± 3.0	71.9 ± 3.8	70.8 ± 3.5	77.0 ± 2.73	79.7 ± 2.9	74.4 ± 3.0	79.7 ± 2.6	72.6 ± 3.8
Raise Forehead Strong	Accuracy	64.6 ± 2.7	76.8 ± 2.6	75.1 ± 2.6	74.8 ± 2.5	79.1 ± 2.9	81.7 ± 2.7	73.3 ± 3.0	81.7 ± 1.8	75.5 ± 2.6
	Precision	53.0 ± 3.9	72.3 ± 2.9	72.7 ± 3.1	71.0 ± 3.3	75.8 ± 3.3	78.3 ± 3.5	71.1 ± 3.4	78.4 ± 2.5	73.2 ± 2.7
	Recall	72.2 ± 5.5	77.6 ± 3.3	73.1 ± 2.9	73.0 ± 2.9	78.3 ± 3.3	81.0 ± 3.0	71.2 ± 3.5	80.9 ± 2.2	73.2 ± 3.0
	F1-score	54.8 ± 4.7	74.2 ± 3.0	72.8 ± 2.9	71.8 ± 3.0	76.8 ± 3.2	79.3 ± 3.2	71.0 ± 3.3	79.4 ± 2.3	73.0 ± 2.8
Raise Forehead Light	Accuracy	55.6 ± 1.7	76.1 ± 2.5	75.1 ± 2.8	74.0 ± 2.4	80.1 ± 2.4	83.3 ± 2.3	73.6 ± 3.0	83.6 ± 2.4	74.7 ± 2.5
	Precision	35.7 ± 3.8	72.2 ± 3.3	71.5 ± 3.5	69.2 ± 3.2	78.6 ± 3.2	81.7 ± 2.6	72.0 ± 3.7	82.2 ± 2.7	73.0 ± 3.1
	Recall	34.1 ± 1.8	77.6 ± 3.1	74.0 ± 3.3	73.5 ± 3.1	79.9 ± 2.7	83.3 ± 2.6	72.6 ± 3.4	83.4 ± 2.9	73.3 ± 2.7
	F1-score	28.1 ± 6.6	74.0 ± 3.1	72.4 ± 3.3	70.8 ± 3.1	79.0 ± 2.8	82.3 ± 2.5	72.1 ± 3.5	82.6 ± 2.7	73.0 ± 2.7

DT: Decision tree, *k*-NN: k-nearest Neighbors, RF: Random forest, SVM: Support vector machine.

**Table 3:** Performance metrics of the decision tree (DT) ensemble and the random forest (100 trees, RF) model, differentiating HB 1 of healthy subjects and patients.

	Metric	Smile Strong	Smile Light	Close Eyes Strong	Close Eyes Light	Raise Forehead Strong	Raise Forehead Light
**RF (100)**	Accuracy (%)	86.9 ± 4.3	88.6 ± 3.5	90.0 ± 3.6	97.2 ± 2.2	85.0 ± 3.9	85.1 ± 3.6
	Precision (%)	86.6 ± 4.6	88.2 ± 3.7	89.6 ± 3.8	97.0 ± 2.2	84.5 ± 4.0	84.9 ± 3.8
	Recall (%)	87.2 ± 4.3	88.8 ± 3.6	90.4 ± 3.5	97.3 ± 2.1	85.4 ± 4.0	85.2 ± 3.6
	F1-score	86.6 ± 4.5	88.3 ± 3.7	89.8 ± 3.7	97.1 ± 2.2	84.7 ± 4.0	84.8 ± 3.8
	AUC HB 1 healthy	0.939	0.954	0.962	0.990	0.928	0.927
	AUC HB 1 patient	0.939	0.954	0.962	0.990	0.928	0.927
**DT Ensemble**	Accuracy (%)	86.1 ± 5.2	88.5 ± 3.7	89.9 ± 3.6	97.2 ± 2.1	84.0 ± 4.8	85.6 ± 4.5
	Precision (%)	85.7 ± 5.3	88.1 ± 3.9	89.4 ± 3.9	97.0 ± 2.3	83.5 ± 4.6	85.5 ± 4.6
	Recall (%)	86.5 ± 5.4	88.6 ± 3.8	90.4 ± 3.4	97.4 ± 1.9	84.7 ± 4.8	85.7 ± 4.4
	F1-score	85.8 ± 5.3	88.2 ± 3.8	89.6 ± 3.8	97.1 ± 2.1	83.6 ± 4.8	85.4 ± 4.6
	AUC HB 1 healthy	0.930	0.952	0.963	0.992	0.926	0.927
	AUC HB 1 patient	0.930	0.952	0.961	0.992	0.926	0.929

## Data Availability

The data presented in this study are available on request from the corresponding author due to ethical reasons.

## Abbreviations

The following abbreviations are used in this manuscript:

ACC	Accuracy
ALS	Amyotrophic lateral sclerosis
ANN	Artificial neural network
AUC	Area under the curve
CNN	Convolutional neural networks
DT	Decision trees
DTE	Decision tree ensemble
ECOC	Error-correcting output codes
EMG	Electromyography
FP	Facial palsy
HB	House–Brackmann score
*k*-NN	k-nearest neighbors
ML	Machine learning
MNLR	Multinomial logistic regression
NCS	Nerve conduction studies
NN	Neural network
QoL	Quality of life
OvR	One-vs-rest
RBF	Radial basis function
RF	Random forests
ROC	Receiver operating characteristic
sEMG	Surface electromyography
SVM	Support vector machines
